# Contextualizing AI-Supported Emotion Regulation Through Sport and Exercise in Higher Education: A Conceptual Reframing

**DOI:** 10.3390/bs16071173

**Published:** 2026-07-11

**Authors:** Yuze Zhang, Syed Ghufran Hadier, Yinghai Liu, Yanlan Guo

**Affiliations:** 1 College of Physical Education, Shanxi University, Taiyuan 030006, China; zhangyuze@sxu.edu.cn; 2 College of Physical Education, North University of China, Taiyuan 030051, China20250073@nuc.edu.cn (Y.G.)

**Keywords:** artificial intelligence, emotion regulation, university students, sport intervention, exercise psychology, higher education, BERTopic, contextual intervention design

## Abstract

Background: Artificial intelligence (AI) is being increasingly used in educational and mental health contexts, yet many emotion-related applications still prioritize detection, classification, and automated feedback over contextual understanding. This study uses the Stanford AI Index Reports (2021–2025) as an exploratory discourse corpus to examine how prominent AI reports frame technology, application domains, and governance, and to consider what this framing implies for emotion regulation through sport and exercise in higher education. Methods: Across 1813 report pages, we applied BERTopic with multilingual sentence embeddings (paraphrase-multilingual-MiniLM-L12-v2), UMAP dimensionality reduction, HDBSCAN clustering, and class-based TF-IDF, followed by dynamic and hierarchical topic analysis and theory-informed synthesis. Of 22 topics generated, 13 relevant to the study focus were retained and validated through keyword inspection, representative-text review, and independent expert agreement. Results: The analysis indicated a three-layer structure: a technology core, an application-expansion layer, and an ethics-and-governance layer. Health and education themes grew most across reports, with medicine/health rising from 14 to 105 and school pathways from 3 to 105 segment occurrences between 2021 and 2025, whereas sport, exercise, embodied activity, and campus support appeared only indirectly. As prominence reflects raw frequency across five reports, trends are read descriptively. Conclusions: We propose a human–technology–environment framework comprising multimodal contextual profiling, autonomy-supportive task adaptation, feedback–reflection–practice loops, peer and campus support integration, and human-in-the-loop governance. The study does not test intervention effects; its contribution is conceptual and agenda-setting, clarifying a gap between mainstream AI discourse and the embodied, relational, and ecological conditions through which sport and exercise may support students’ emotion regulation.

## 1. Introduction

The mental health of university students remains a pressing concern for higher-education systems. Large international surveys and recent meta-analyses show that substantial proportions of students experience depression, anxiety, stress, or other clinically relevant psychological difficulties, and that these difficulties are shaped by academic workload, social relationships, institutional climate, economic pressure, and access to support (Auerbach et al., 2018; Sheldon et al., 2021; W. Li et al., 2022). Digital mental health interventions have expanded rapidly in response to this demand, and recent evidence suggests that such interventions can reduce depressive and anxiety symptoms among university students, although engagement, intervention fidelity, contextual fit, and evaluation quality remain uneven (Madrid-Cagigal et al., 2025). These findings indicate that the key challenge is no longer simply whether students need support, but how support can be designed so that it is timely, acceptable, contextually meaningful, and ethically responsible.

Emotion regulation provides an important theoretical lens for addressing this challenge. It refers to processes through which individuals influence the occurrence, intensity, duration, expression, or meaning of their emotional responses (Gross, 2015; McRae & Gross, 2020). In university settings, emotion regulation is not only an individual cognitive skill. It is also embedded in students’ daily routines, course pressures, peer interactions, bodily states, and available institutional resources. Recent work on emotion regulation in higher-education populations reinforces this situated view, showing that students’ regulatory difficulties are bound up with academic stressors, social environments, and daily routines rather than reflecting a stable individual trait (Brites et al., 2024). This point is central for the present study because AI-supported emotion regulation will remain limited if it focuses only on detecting emotional states or delivering standardized advice. A more adequate approach must consider how students understand, practice, and sustain regulation within real educational, social, and physical activity (PA) contexts.

AI has created new opportunities for mental health and educational support, especially through affective computing, multimodal emotion recognition, learning analytics, recommender systems, and conversational agents. Systematic reviews show rapid technical progress in recognizing affective signals from text, speech, facial expression, physiological data, and behavioral traces (Khare et al., 2024; W. Zhang et al., 2022). AI-based conversational agents have also shown potential for reducing psychological distress, while large language model applications in mental health care are increasingly explored for screening, counseling, psychoeducation, and emotional support (H. Li et al., 2023; Hua et al., 2025). However, these developments also expose a major limitation: emotional data do not speak for themselves. Psychological evidence shows that emotional states cannot be reliably inferred from a single expressive channel without contextual information (Barrett et al., 2019), while work on emotional AI cautions that affect-sensing systems raise distinct privacy and surveillance concerns when applied to everyday emotional life (McStay, 2020). Emotion recognition can be biased, culturally variable, privacy-sensitive, and difficult to interpret when detached from context (Barrett et al., 2019; McStay, 2020). Therefore, the central question is not only whether AI can classify emotion more accurately, but whether it can support human judgment and context-sensitive intervention design without reducing students to data profiles.

Sport and exercise settings are especially relevant to this debate. PA is not merely a lifestyle variable that can be appended to digital mental health support; it can function as a situated context in which students experience bodily activation, challenge, recovery, social contact, competence, enjoyment, and reflective practice (M. Z. H. Hamdani et al., 2022; Long et al., 2026). Recent reviews and meta-analyses support the potential of PA and exercise interventions for improving undergraduate mental health and depressive symptoms, while also emphasizing heterogeneity in effects and the need for stronger theory-informed implementation (Huang et al., 2024; Noetel et al., 2024; Yang et al., 2025; Liu et al., 2025). A best-evidence synthesis further suggests that affect, self-efficacy, physical self-worth, resilience, social support, and social connection are plausible mediators linking PA to mental health outcomes (White et al., 2024). Beyond symptom reduction, exercise has been linked specifically to emotion-regulation capacity: regular PA is associated with greater use of adaptive regulation strategies and may buffer difficulties in down-regulating negative affect (Bernstein & McNally, 2018). These findings align with sport and exercise psychology perspectives that treat movement, task constraints, motivation, and social participation as active mechanisms rather than neutral delivery channels (Bernstein & McNally, 2018; Ntoumanis et al., 2021; Rhodes et al., 2019).

Despite these converging lines of evidence, AI-informed mental health research and sport/exercise-based emotion regulation research remain insufficiently integrated. Much AI-related work still privileges detection, classification, prediction, or automated feedback, whereas much exercise-based mental health work focuses on intervention effects, adherence, dose, and psychosocial mechanisms without specifying how AI should be responsibly embedded in physical education (PE), campus sport, counseling, or peer-support systems. This gap is theoretical as well as practical. If AI is placed at the center of intervention design, PA may be reduced to a recommendation generated after an emotional label is detected. If sport and exercise psychology is placed at the center, AI can be repositioned as a supporting tool for understanding context, adapting tasks, prompting reflection, coordinating campus support, and maintaining human oversight.

Against this background, the present study uses the Stanford AI Index Reports from 2021 to 2025 as an influential but deliberately bound AI discourse corpus. These reports are widely cited annual syntheses that track how the AI field itself frames technical progress, application domains, and governance priorities; they are therefore well suited to revealing what mainstream AI discourse foregrounds and what it leaves implicit, even though they do not represent the full research literature on emotion, sport, or student mental health. The purpose is not to conduct an intervention trial, a systematic review of all AI-supported sport interventions, or a meta-analysis of exercise effects. Rather, the study combines BERTopic-informed document analysis with theory-informed conceptual synthesis to examine how major AI discourse frames technology, application, education, health, and governance, and to consider what this framing implies for sport- and exercise-based emotion regulation in higher education. Specifically, the study asks: (1) What thematic structure is visible in the AI Index Reports from 2021 to 2025? (2) How can this structure, interpreted cautiously alongside sport and exercise psychology, inform a human–technology–environment framework for AI-supported emotion regulation in higher education? By making this boundary explicit, the article contributes a conceptual reframing rather than a claim of demonstrated intervention effectiveness.

## 2. Conceptual Background: AI, Emotion Regulation, and Sport Contexts

### 2.1. From Emotion Detection to Contextual Interpretation

Building on the problem outlined above, this section develops the conceptual argument that distinguishes what AI can *detect* from what emotion regulation actually requires. AI-supported emotion regulation is often discussed through the language of detection: systems are expected to identify affective states from text, voice, facial movement, physiological signals, posture, or behavioral traces and then generate feedback or recommendations. This detection-oriented logic has a strong technical basis. Affective computing has progressed from early unimodal models of affect detection (Calvo & D’Mello, 2010; D’Mello & Kory, 2015) toward increasingly multimodal systems that combine verbal, visual, acoustic, physiological, and interactional data (Poria et al., 2017; W. Zhang et al., 2022). In parallel, recent reviews show that automated emotion recognition is expanding in education and online learning environments, while AI-based conversational agents and large language models are increasingly being explored for mental health support (H. Li et al., 2023; Hua et al., 2025; Yu et al., 2024). These developments create practical opportunities for scalable screening, timely feedback, and personalized support in higher education.

The conceptual limit of this approach, however, lies less in technology than in what is left uninterpreted. The central limitation of detection-oriented AI is not simply technical error; it is contextual under interpretation, meaning the treatment of an emotional signal as if its meaning were fixed independently of the situation that produced it. Emotional signals do not carry stable meanings independent of person, task, culture, and situation. Facial movements, voice tone, or text sentiment may indicate distress, effort, embarrassment, fatigue, concentration, social discomfort, or strategic self-presentation depending on the setting. Psychological evidence cautions against assuming that emotion can be directly inferred from a single expressive channel without contextual information (Barrett et al., 2019). For university students, this problem is especially important because emotional episodes often emerge from overlapping academic, interpersonal, bodily, and institutional conditions. A student who appears anxious in a sport class may be responding to performance evaluation, peer comparison, unfamiliar movement demands, physical fatigue, fear of injury, or pressures outside the class. An AI system that classifies the visible signal but ignores these surrounding conditions risks producing recommendations that are technically plausible but interventionally inappropriate.

For this reason, the present study treats AI as a contextual support tool rather than an autonomous interpreter of students’ inner states. Four senses of “support” are used consistently throughout the paper, refers to the interpretation of emotional information in relation to task demands, bodily experience, social relations, place, timing, and student agency. Embodied support means that emotion regulation is considered through action, arousal, interoception, and felt experience, not only through verbal reflection or digital feedback. Socially integrated support means that peer climate, teacher–student interaction, group belonging, and campus services are included in the intervention ecology. Ecologically grounded support means that design decisions are organized around the interaction among person, task, and environment rather than around an isolated user–system dyad.

### 2.2. Emotion Regulation as a Situated and Motivated Process

Emotion regulation is commonly defined as the process by which individuals influence which emotions they have, when they have them, and how they experience or express them (Gross, 2015; McRae & Gross, 2020). The process model of emotion regulation remains useful because it distinguishes families of strategies such as situation selection, situation modification, attentional deployment, cognitive change, and response modulation. Yet emotion regulation should not be understood only as the individual selection of cognitive strategies. Research on emotion-regulation flexibility, motives, and psychopathology shows that the adaptiveness of a strategy depends on goals, context, timing, intensity, and available resources (Aldao et al., 2010; Tamir, 2016). Suppression, avoidance, reappraisal, acceptance, problem solving, and help seeking do not have fixed psychological value across all circumstances; their effects depend on why they are used and whether they fit the situation.

This situated view is directly relevant to university mental health. Students’ emotional regulation demands are distributed across classrooms, dormitories, peer networks, examination periods, digital environments, sport clubs, and counseling systems. Therefore, AI-supported emotion regulation should not be reduced to detecting an emotional label and delivering a standardized suggestion. It should help students and practitioners understand the conditions under which emotions arise, the regulatory resources available in that moment, and the forms of support that preserve students’ autonomy. This distinction also separates the present conceptual framework from a purely clinical or diagnostic model. The aim is not to use AI to diagnose mental disorders or replace psychological services, but to examine how AI-informed systems may support everyday regulation, reflection, and coordinated care in educational and sport contexts.

### 2.3. Sport and Exercise as Embodied Regulation Contexts

Sport and exercise provide distinctive conditions for emotion regulation because they are embodied, task-based, affectively charged, and socially organized. Physical activity can influence mental health through physiological arousal and recovery, neurobiological pathways, attentional redirection, perceived competence, mastery experiences, identity formation, and social connection (Bernstein & McNally, 2018; Kandola et al., 2019; Lubans et al., 2016; White et al., 2024). Meta-analytic and review evidence has linked PA and exercise with lower depressive and anxiety symptoms, including among university populations, although effect sizes and mechanisms vary by population, activity type, intensity, adherence, and intervention design (Huang et al., 2024; Liu et al., 2025; Noetel et al., 2024; Rebar et al., 2015). Thus, exercise should not be framed as a generic behavioral prescription, but as a structured context in which affective experience can be generated, interpreted, and practiced.

The affective character of exercise is particularly important. Students do not respond only to the amount of activity prescribed; they respond to how the activity feels, whether it is perceived as meaningful or threatening, whether challenge is calibrated, and whether participation supports or undermines competence and autonomy (Hadier et al., 2024a, 2024b; S. M. Z. H. Hamdani et al., 2023; Yang et al., 2025). The affective–reflective theory of physical inactivity and exercise argues that automatic affective valuations and reflective evaluations jointly shape activity behavior (Brand & Ekkekakis, 2018). Self-determination theory similarly suggests that sustained engagement depends on autonomy, competence, and relatedness, and empirical reviews have applied these principles to exercise and health behavior change (Ryan & Deci, 2000, 2020; Teixeira et al., 2012; Ntoumanis et al., 2021). These perspectives imply that AI-supported exercise recommendations should not simply increase activity volume; they should help teachers and students adjust task difficulty, choice, feedback, grouping, and progression so that regulation practice remains psychologically safe and motivationally sustainable.

### 2.4. Sport Contexts as Social and Ecological Systems

Sport and exercise settings are also social ecologies. A university physical education class, a campus running group, a martial arts course, or a recreational sport club involves norms, roles, feedback, competition, cooperation, and shared emotional rhythms. These features can strengthen belonging and regulation resources, but they can also intensify social comparison, embarrassment, exclusion, or performance anxiety if poorly designed. Ecological systems theory reminds us that student wellbeing is shaped by multiple levels of context, from immediate interpersonal settings to institutional culture and policy (Bronfenbrenner, 1979). In sport pedagogy, ecological dynamics and the constraint-led approach further emphasize that behavior emerges from the interaction of individual, task, and environmental constraints rather than from isolated internal traits (Renshaw & Chow, 2019; Renshaw et al., 2019; Woods et al., 2020).

This ecological view clarifies what AI can and cannot contribute. AI may help summarize participation patterns, detect changes in engagement, identify mismatches between task demand and student response, or provide reflective prompts after practice. It may also help teachers, coaches, counselors, and sport psychologists coordinate support when repeated patterns of distress or withdrawal appear. Yet these functions are useful only when embedded in human judgment and institutional care. If AI converts social–emotional data into surveillance, ranking, or compulsory behavioral correction, it may undermine precisely the autonomy and relational safety that sport-based emotion regulation requires. Therefore, the design question is not whether AI can enter sport and exercise contexts, but under what governance conditions it can support student agency, embodied learning, and ethically responsible care.

### 2.5. Toward a Human–Technology–Environment Perspective

The conceptual position developed in this paper is a human technology environment perspective. This perspective does not reject AI; it repositions AI. Within this perspective, students, physical education teachers, coaches, counselors, and sport psychologists remain the primary agents of interpretation, care, and ethical judgment, while AI functions as a supporting tool for pattern recognition, documentation, visualization, and timely prompts. Technology provides pattern recognition, visualization, documentation, and timely prompts. The environment supplies the task structures, peer relations, physical spaces, institutional routines, and governance arrangements through which emotion regulation is practiced. The value of AI-supported sport and exercise interventions therefore depends on the quality of alignment among these three elements.

This perspective also sets the evidential boundary of the present study. The BERTopic analysis of the Stanford AI Index Reports can reveal how mainstream AI discourse organizes themes of technology, application, and governance. It cannot directly prove that AI-supported sport and exercise interventions improve emotion regulation outcomes. The role of the conceptual background is therefore to build a theoretically grounded bridge between the AI discourse analyzed in the corpus and the sport/exercise psychology framework developed later in the paper. This bridge is necessary because emotionally sensitive AI systems require more than accurate detection: they require contextual interpretation, student agency, participatory design, data minimization, bias awareness, transparency, and human oversight (Jobin et al., 2019; McStay, 2020; Mittelstadt, 2019).

## 3. Materials and Methods

### 3.1. Design Rationale and Analytical Positioning

The study used a document-based, mixed analytic design combining computational text analysis with theory-informed qualitative interpretation. The overall logic was to move from corpus construction to topic extraction and structural analysis, and then to conceptual synthesis for sport and exercise psychology. The aim was not to conduct a systematic review or meta-analysis of intervention effects. Rather, the study examined how major AI developments were represented across a prominent annual AI corpus and how those patterns could inform a context-sensitive model of AI-supported sport intervention for emotion regulation in university settings.

This approach is consistent with a growing body of work that applies computational topic modeling to large policy, scientific, and institutional document collections in order to characterize how a field frames its priorities over time. Where earlier studies of this kind have often relied on bag-of-words models such as Latent Dirichlet Allocation, the present study uses a transformer-embedding approach (BERTopic) that is better suited to short, heterogeneous text segments and preserves more semantic context (Grootendorst, 2022). In positioning the design this way, we treat the computational analysis as a structured reading of discourse rather than as hypothesis-testing, which aligns the method with its conceptual, agenda-setting purpose and distinguishes it from intervention-effect or systematic-review designs.

### 3.2. Core Corpus: Stanford AI Index Reports, 2021–2025

The core corpus comprised the Stanford AI Index Reports from 2021, 2022, 2023, 2024, and 2025 (Maslej et al., 2023, 2024, 2025; D. Zhang et al., 2021, 2022). These reports were selected for three reasons. First, they are influential annual syntheses of AI development and are frequently used by policy, industry, and academic audiences. Second, they cover multiple domains relevant to the present topic, including technical performance, education, health, public opinion, policy, responsible AI, and governance. Third, their annual format allowed descriptive comparison across five reports. The five working PDF files contained 1813 pages in total. After extraction and cleaning, the analysis was organized around textual segments derived from paragraphs and coherent text blocks rather than complete pages or entire chapters.

The corpus should be interpreted narrowly. It represents a high-level AI discourse corpus, not the full literature on AI, student mental health, emotion regulation, sport psychology, or exercise intervention. This boundary is important because conclusions about the absence or underdevelopment of sport and exercise themes refer to the AI Index corpus only.

### 3.3. Theory-Supporting Literature and Interpretive Boundaries

Supporting studies in the literature were used to interpret the topic-modeling output and to develop the sport and exercise psychology framework. It was not pooled into the BERTopic corpus. The supporting literature was identified through targeted searches of Web of Science, PubMed, PsycINFO, Google Scholar, and CNKI using combinations of terms related to AI emotion recognition, affective computing, student mental health, physical activity interventions, sport and exercise psychology, ecological dynamics, self-determination theory, social support, and AI ethics. Priority was given to recent systematic reviews, meta-analyses, conceptual papers, and widely used theoretical sources. Because these studies were used for conceptual interpretation rather than for effect-size synthesis, review quality tools such as AMSTAR 2, ROBIS, or JBI SUMARI were not applied.

### 3.4. Text Extraction, Cleaning, and Paragraph Segmentation

PDF files were converted to machine-readable text. Repeated headers and footers, isolated page numbers, copyright notes, broken hyphenation, non-substantive symbols, and very short fragments were removed. Report sections, figure notes, and table descriptions were retained when they contained substantive content. Text was segmented into semantically coherent units using paragraph breaks where available and line/block-level segmentation where the PDF structure did not preserve paragraphs. Segments consisting only of numerals, navigation elements, or disconnected captions were excluded. The cleaned segments formed the analytic units subsequently embedded and clustered in the BERTopic procedure (Section 3.5). Annual report identifiers were retained so that descriptive topic prominence could be summarized by year.

### 3.5. BERTopic Modeling Procedure and Parameter Settings

BERTopic is an embedding-based topic-modeling approach. It first transforms each text unit into a semantic vector using a transformer language model, then reduces the high-dimensional vectors, clusters semantically similar units, and uses class-based term frequency-inverse document frequency (c-TF-IDF) to generate interpretable topic words (Grootendorst, 2022). This differs from classical bag-of-words models such as LDA, which primarily rely on word co-occurrence. BERTopic was selected because the AI Index corpus is heterogeneous and contains technical, policy, educational, and health-related language across multiple years.

The operational workflow proceeded in six steps. First, text embedding was performed by invoking the paraphrase-multilingual-MiniLM-L12-v2 model to convert each paragraph of the preprocessed corpus into a high-dimensional semantic vector; a multilingual model was used to accommodate mixed-language technical terminology in the source material. Second, dimensionality reduction was conducted using Uniform Manifold Approximation and Projection (UMAP), which maps the high-dimensional vectors into a low-dimensional space while preserving key semantic structures to facilitate clustering. Third, clustering was executed by applying Hierarchical Density-Based Spatial Clustering of Applications with Noise (HDBSCAN) to the reduced vectors; this algorithm identifies clusters of varying shape and density, automatically detects noise points, and groups semantically similar segments into the same thematic cluster. Fourth, topic representation was carried out using class-based TF-IDF (c-TF-IDF), which weights each word within a cluster relative to the entire corpus to generate the keyword list that best characterizes each topic. Fifth, topic evolution was examined using BERTopic’s dynamic topic-modeling functionality, segmenting the corpus by report year (2021–2025) to calculate the distribution of topics across periods and to visualize shifts in prominence over time. Sixth, topic relationships were examined by applying hierarchical clustering (via the scipy.cluster.hierarchy module) to all generated topics, producing a topic relationship map that was used to assess the rationale for the higher-order dimensional grouping and to reveal latent associations among topics.

The procedure initially produced 22 topics. After excluding clusters unrelated to the study focus and retaining those relevant to sport, exercise, education, and health-related discourse, 13 topics were carried forward for interpretation. All analyses were conducted in Python v3.11.9 using BERTopic v0.17.0, together with the associated sentence-transformers, UMAP, HDBSCAN, and scikit-learn libraries. Because unsupervised topic models are sensitive to preprocessing and clustering choices, the results are interpreted descriptively rather than inferentially. The reporting details for the computational procedure are summarized in Table 1.

### 3.6. Topic Interpretation, Validation, and Robustness Checks

Topic labels were not assigned mechanically from the single highest-weight word. Instead, labels were developed by considering the top c-TF-IDF words, representative-text units, report-year distribution, and the location of the material within the original reports. Two researchers with backgrounds in sport psychology and educational technology independently reviewed preliminary labels. Disagreements were resolved through discussion, with attention to whether a label was too broad, too technical, or too interpretive. When topics were conceptually adjacent, labels were kept conservative. For example, topics containing medical, healthcare, and patient terms were labeled medicine/health rather than mental health, unless the representative text directly concerned mental health.

**Figure 1:**
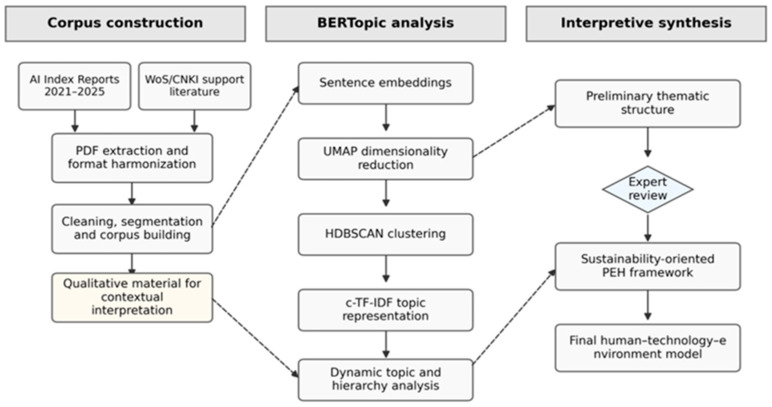
Research design and analytic workflow.

Topic validation was treated as a triangulation process. Automated topic indicators are useful but insufficient for unsupervised neural topic models because coherence measures may not always align with human interpretability (Hoyle et al., 2021). We therefore emphasized label transparency, representative-text inspection, expert review, and cautious interpretation. This strategy strengthens trustworthiness without overstating the statistical status of the topics. The complete research design and analytic workflow are summarized in Figure 1.

## 4. Results

### 4.1. Thematic Structure: Technology, Application, and Governance Layers

Table 2 summarizes the retained topics, their representative keywords, and the interpretive boundary attached to each. As shown in the table, the topics fall into four interpretive groupings that together describe the corpus structure. The technology-core topics (training/data, computing disciplines, and AI performance and reports) are dominated by benchmarking, infrastructure, and computational vocabulary, indicating that the AI Index discourse foregrounds technical capability rather than emotional or contextual understanding. The application-expansion topics form the largest grouping and span medicine/health, higher education, computer science education, school pathways, degree growth, skills/jobs, economy/industry, and IT or assistive design; these topics show that AI is increasingly discussed in relation to health, education, and the labor market, but the representative keywords remain institutional and workforce-oriented rather than concerned with student wellbeing, embodied experience, or peer climate. The ethics-and-governance topics (ethics and research/government coordination) capture privacy, accountability, oversight, and policy framing, confirming that responsible-AI concerns are present in the discourse. The final row records what the corpus does not articulate as a coherent theme: sport, exercise, peer interaction, and campus ecology appear only diffusely, distributed across health- and education-related fragments rather than forming a distinct topic. The “interpretive boundary” column is included to prevent overreading; for each topic, it states what the keywords can and cannot support, so that, for example, the presence of a medicine/health topic is not mistaken for evidence of attention to student mental health or sport-based emotion regulation specifically.

**Figure 2:**
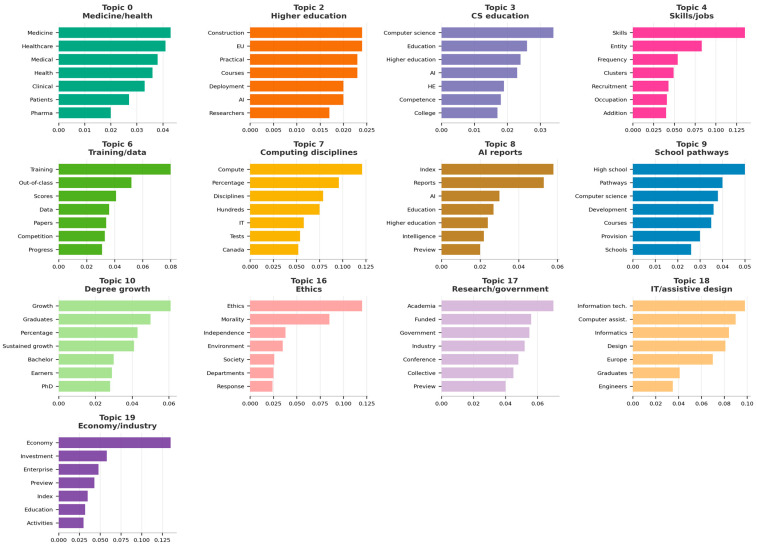
Topic–word distribution extracted from the AI Index corpus.

The topic-modeling procedure produced 22 initial topics, of which 13 were retained after excluding clusters unrelated to the study focus (Section 3.5). These 13 retained topics are presented in Figure 2, which displays, for each topic, the highest-weighted keywords ranked by their c-TF-IDF score; the score on the horizontal axis indicates how strongly each word characterizes that topic relative to the rest of the corpus. Reading the topics together, the retained structure was organized around three broad layers. The first was a technology core, including training/data, AI performance, computing disciplines, and model infrastructure (e.g., Topics 6, 7, and 8). The second was an application-expansion layer, including medicine/health, higher education, computer science education, school pathways, skills/jobs, degree growth, economy/industry, and IT or assistive design (e.g., Topics 0, 2, 3, 4, 9, 10, 18, and 19). The third was an ethics-and-governance layer, including ethics, research/government coordination, and policy (e.g., Topics 16 and 17).

This pattern is important for the present study because it shows that education and health are visible within mainstream AI discourse, whereas sport and exercise appear only indirectly, usually through health, education, or physical-activity-related fragments rather than as a coherent intervention context. The keyword profiles in Figure 2 reinforce this reading: even the most application-oriented topics are dominated by institutional, technical, and workforce vocabulary, with no topic foregrounding embodied activity, peer climate, or campus wellbeing. Consistent with the validation strategy described in Section 3.6, each label was checked against its representative keywords and text units, so the three-layer interpretation reflects triangulated reading rather than the single highest-weight word.

### 4.2. Semantic Similarity: Tightly Connected but Unevenly Contextualized Themes

**Figure 3:**
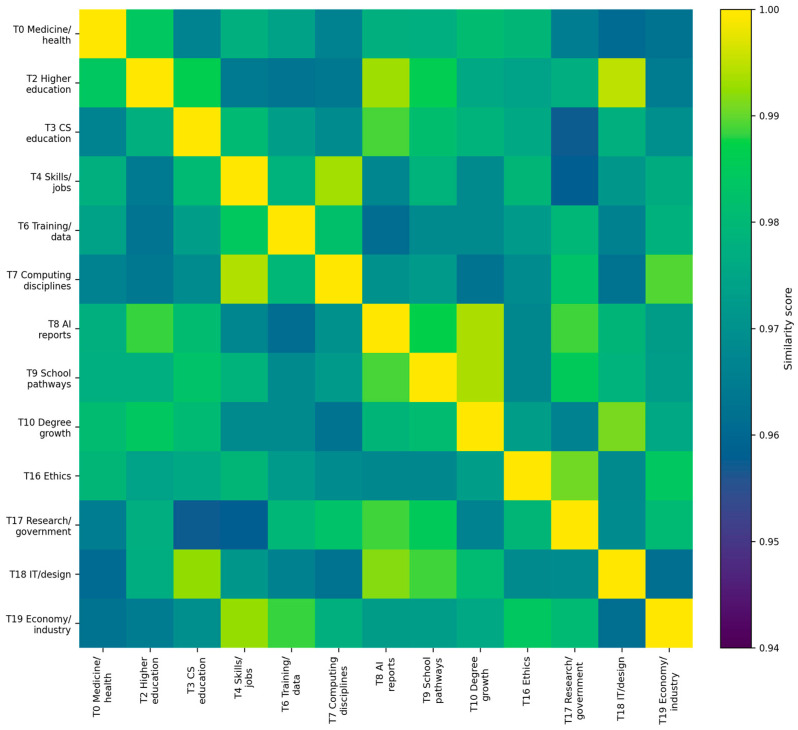
Semantic similarity heatmap of identified topics.

The semantic similarity heatmap (Figure 3) indicated that many topics were closely related, particularly those linking technical capacity, education, and health-related applications. This supports a cautious interpretation: AI discourse increasingly connects technical development with real-world application domains, so the technology core and the application layer are not separate conversations but closely associated ones. However, the similarity structure did not position sport, exercise, peer climate, or campus ecological support as central organizing themes. The implication is not that these topics are absent from all research, but that they are weakly articulated within this high-level AI discourse corpus, which is consistent with the keyword evidence in Figure 2.

### 4.3. Dynamic Topic Trends: Rising Salience of Health and Educational Applications

**Figure 4:**
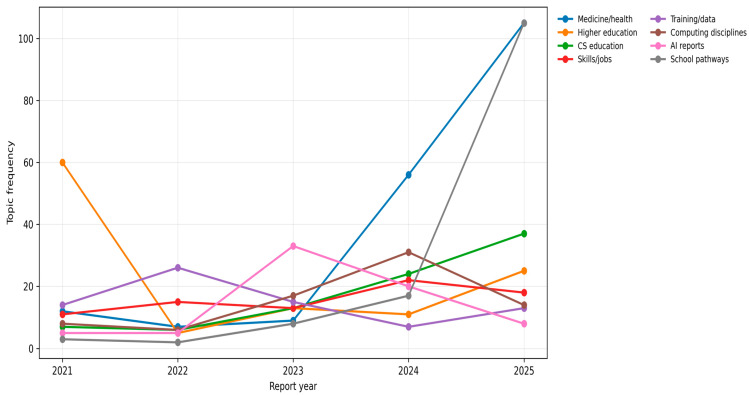
Topic evolution across the AI Index reports (2021–2025).

The dynamic topic analysis (Figure 4) described shifts in topic prominence across the 2021–2025 reports. In this analysis, prominence is expressed as the raw frequency of segments assigned to each topic within each report year, rather than as a normalized proportion. Health-related and school-pathway topics rose markedly in the later reports, with medicine/health and school pathways increasing sharply in 2024–2025, whereas higher education and several technology-oriented topics fluctuated without a consistent direction.

These patterns should be interpreted cautiously for two reasons. First, the corpus includes only five annual reports, which is insufficient for formal trend inference. Second, because prominence is measured as raw frequency, apparent increases may partly reflect report length, the addition of new chapters, or changes in report structure and editorial priorities rather than changes in the AI field as a whole. The dynamic analysis is therefore used only to describe shifts within the report series, not to model the trajectory of AI research generally.

For the present argument, the robust point is narrower: across the five reports, health and education became more visible than sport or exercise as explicit contexts for AI-supported emotion regulation. This descriptive finding motivates, but does not prove, the need for a sport and exercise psychology framework.

### 4.4. Hierarchical Structure: From Technical Infrastructure to Context-Relevant Implications

**Figure 5:**
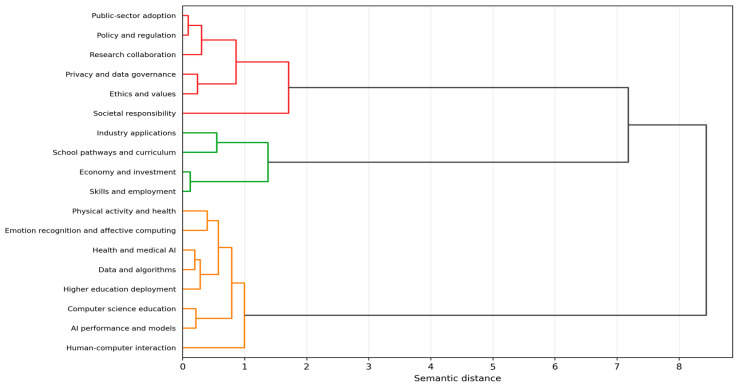
Hierarchical clustering of topic representations.

The hierarchical clustering result (Figure 5) supported the three-layer interpretation described above. Topics concerning data, algorithms, AI performance, and computing clustered near the technology core. Education, health, industry, and assistive design formed application-oriented branches. Ethics, policy, public-sector adoption, and research/government coordination appeared as governance-oriented branches. This structure consists of the broader movement of AI from technical capability toward deployment and social responsibility.

Taken together, the results speak to the second research objective, which asked how this thematic structure might inform a framework for AI-supported emotion regulation in higher education. The corpus shows that AI discourse has technical foundations, an application reach into health and education, and the governance vocabulary needed to support such work, but it does not yet articulate sport, exercise, embodiment, peer climate, or campus ecology as explicit contexts for emotion regulation. The contribution of the following framework is therefore to connect the capabilities visible in this discourse to the embodied task design, autonomy support, social connection, and ethical oversight that sport- and exercise-based emotion regulation requires. This is an interpretive bridge from the corpus to theory, not a demonstrated intervention effect.

## 5. Discussion

This study examined the Stanford AI Index Reports as an exploratory corpus and used the resulting topic structure to inform a sport and exercise psychology framework for AI-informed emotion regulation support in higher education. The main contribution is conceptual and agenda-setting. The BERTopic analysis identifies what is prominent within the AI Index discourse: technology, expanding applications, and governance.

### 5.1. What the AI Discourse Reveals for Emotion-Recognition Research

The findings align with reviews showing that AI emotion-recognition research has advanced technically but continues to face challenges related to context, generalizability, multimodal ambiguity, and ethical deployment (Khare et al., 2024; Yu et al., 2024). The AI Index corpus reflects this broader field orientation: performance, datasets, deployment, and governance are strongly represented, whereas the lived ecology of emotional support is less developed. This convergence is informative. The three-layer structure observed here (technology core, application expansion, and governance) mirrors the trajectory that prior critiques of emotion AI have described, in which technical capability and benchmarking mature faster than contextual interpretation and ethical integration (Barrett et al., 2019; McStay, 2020). The corpus evidence therefore supports the manuscript’s main conceptual claim that emotion-related AI in higher education should move beyond the detection-recommendation model.

### 5.2. Why Sport and Exercise Psychology Extends the AI Discussion

The discussion situates the argument within physical activity and student wellbeing research. Meta-analytic evidence suggests that physical activity can improve undergraduate mental health outcomes, but the evidence base is heterogeneous and sometimes limited by low certainty, intervention-fidelity problems, and inconsistent theoretical grounding (Huang et al., 2024). This is precisely why a contextual framework is needed. The framework should not ask only whether students exercise more. It should ask how activity tasks, social climates, autonomy support, and feedback structures shape emotion-regulation practice over time.

Self-determination theory explains the motivational conditions under which students are more likely to internalize and sustain activity participation: autonomy, competence, and relatedness (Ryan & Deci, 2000, 2020). Ecological dynamics describe task–environment logic: emotional and behavioral responses emerge as students interact with constraints, affordances, and feedback in real activities (Renshaw & Chow, 2019; Woods et al., 2020). Social–ecological perspectives describe the broader campus level: students’ distress and regulation opportunities are shaped by academic routines, peer networks, institutional culture, and access to support services (Bronfenbrenner, 1979; Roy et al., 2025).

### 5.3. Reframing AI-Supported Emotion Regulation: The Human–Technology–Environment Framework

The proposed framework contains five pathways. These pathways should be read as design principles requiring empirical testing, not as established causal mechanisms. Their practical implementation is summarized in Table 3.

1. Multimodal contextual profiling: AI may integrate self-report, exercise participation, subjective exertion, workload, sleep or recovery indicators, and contextual notes, but data collection should be limited to what is necessary and consented.
2. Autonomy-supportive task adaptation: AI may help practitioners adapt intensity, task complexity, group size, feedback frequency, and recovery options without turning exercise into algorithmic prescription or coercive monitoring.
3. Feedback–reflection–practice loops: AI visualizations may support students in noticing patterns between activity, stress, mood, and coping, but feedback should scaffold self-regulation rather than replace student judgment.
4. Peer and campus support integration: AI tools should be embedded in physical education, sport clubs, counseling services, peer-led activities, and referral pathways instead of remaining isolated individual apps.
5. Human-in-the-loop governance: Teachers, coaches, counselors, and sport psychologists should retain responsibility for interpreting AI outputs, identifying misclassification risks, and protecting students from harmful or stigmatizing feedback.

### 5.4. Responsible Implementation in University Sport and Exercise Contexts

Ethical governance is central because the proposed area combines emotional data, student populations, educational authority, and behavior guidance. The ethical discussion therefore focuses on seven issues: (1) privacy: emotional, behavioral, and physiological data should be treated as sensitive; (2) consent: students should know what is collected, why it is collected, and how it will be used; (3) data minimization: systems should avoid collecting data that are not needed for the stated support function; (4) transparency: students and practitioners should understand the uncertainty and limitations of AI outputs; (5) bias: models may misread emotion across gender, culture, disability, language, and neurodiversity; (6) autonomy: recommendations should not become coercive exercise prescriptions; (7) human oversight: AI outputs should support, not replace, professional and pedagogical judgment.

These safeguards also prevent a common failure mode in emotion AI, the transformation of support into surveillance. In university sport and exercise contexts, monitoring can easily become coercive if emotional analytics are tied to attendance, performance grading, disciplinary decisions, or institutional risk management. A credible AI-informed framework must therefore begin with governance before moving to technical optimization. Supplementary materials consist of the Stanford AI Index reports information.

### 5.5. Limitations of Corpus, Method, and Conceptual Inference

This study has several limitations. First, the corpus is narrow. The AI Index Reports are influential, but they do not represent the full AI literature, sport and exercise psychology literature, student mental health literature, or intervention research. Second, topic modeling is exploratory and sensitive to segmentation, preprocessing, embedding models, dimensionality reduction, clustering parameters, and topic-reduction decisions. Third, the dynamic analysis is based on only five annual reports and should not be interpreted as a formal longitudinal trend analysis. Fourth, the framework development is interpretive. The five pathways were informed by the topic model but were mainly developed through theory-informed synthesis. Fifth, the study did not test intervention effects. It provides no direct empirical evidence that AI-supported sport or exercise interventions improve emotion regulation outcomes. Sixth, the supporting literature was not reviewed systematically, so the discussion should be read as conceptual integration rather than exhaustive evidence synthesis.

### 5.6. Future Research

Future research should move from conceptual design to empirical testing. Longitudinal intervention studies could examine whether AI-supported feedback helps students develop emotion-regulation skills across a semester. Randomized controlled trials could compare standard physical activity programs with autonomy-supportive, AI-informed task adaptation. Mixed-methods studies could combine mood and activity data with interviews to understand how students experience feedback, privacy, and peer climate. Participatory design research could involve students, teachers, counselors, and AI designers in building tools that are acceptable and ethically credible. Comparative studies across universities could examine how institutional culture, digital readiness, and campus support structures shape implementation. Mechanistically, future work should test how autonomy support, peer climate, task constraints, feedback timing, and perceived surveillance interact over time.

## 6. Conclusions

This study reaches a deliberately modest conclusion. The AI Index Reports from 2021 to 2025 show a broad AI discourse organized around technical capacity, application expansion, and ethical governance. Within this corpus, education and health are visible, but sport, exercise, peer interaction, and campus ecology are not developed as central contexts for emotion regulation support. This does not mean that the wider literature has ignored these issues. It means that they are underrepresented in this prominent AI discourse corpus and therefore require stronger conceptual translation from sport and exercise psychology.

The originality of the study lies in translating this gap into a sport and exercise psychology agenda. Rather than treating AI as the intervention itself, the paper repositions it as a means of supporting contextual understanding, autonomy-supportive task adaptation, reflective practice, peer and campus support, and human-governed decision-making. In doing so, the study answers its two guiding objectives: it characterizes the thematic structure of a major AI discourse corpus, and it uses that structure, interpreted alongside sport and exercise psychology, to derive a human–technology–environment framework for AI-supported emotion regulation in higher education. The framework offers design principles for future empirical research rather than evidence of demonstrated effectiveness, and its value will ultimately depend on the rigor of the intervention studies it is intended to motivate.

## Figures and Tables

**Table 1:** Reporting details for the computational procedure.

Analytic Component	Information Now Clarified	Purpose of Clarification
Corpus	Five Stanford AI Index Reports, 2021–2025; total working file length: 1813 PDF pages.	Justifies the corpus as broad AI discourse rather than sport-specific evidence.
Unit of analysis	Cleaned textual segments derived from paragraphs or coherent PDF text blocks.	Prevents ambiguity between page-, paragraph-, and chapter-level analysis.
Preprocessing	Removal of headers, footers, page labels, isolated numerals, broken symbols, and duplicated navigation text.	Improves reproducibility and reduces non-substantive topic noise.
BERTopic logic	Transformer embeddings -> UMAP -> HDBSCAN -> c-TF-IDF -> topic labeling.	Explains BERTopic for readers unfamiliar with the method.
Validation	Topic labels checked through keyword consistency, representative-text inspection, expert review, and sensitivity-oriented interpretation.	Avoids treating unsupervised topics as self-validating.
Interpretive boundary	Corpus results inform the framework but do not empirically prove intervention effects.	Addresses the main concern about overclaiming.

**Table 2:** Summary of major topics and interpretive boundaries.

Layer	Topic Label	Topic Size, n (%)	Representative Keywords	Interpretive Boundary
Technology core	AI Index reports/discourse; computing disciplines	141 (4.8%);	training, data, compute, tests, index, reports	Shows the technical and benchmarking emphasis of the AI discourse.
Technology core	AI performance and models	95 (3.2%)	models, benchmarks, language, multimodal, performance	Supports claims about technical capability, not emotional understanding.
Application expansion	Medicine/health	172 (5.8%)	medicine, healthcare, medical, health, clinical, patients	Indicates health relevance, but not necessarily sport or student mental health.
Application expansion	Higher education	82 (2.8%)	higher education, courses, deployment, researchers, students	Indicates educational application, but not automatically campus wellbeing.
Application expansion	Computer science education	78 (2.6%)	computer science, education, competence, college, AI	Shows education and skills discourse, mostly technology education.
Application expansion	School pathways and degree growth	79 (2.7%)	high school, pathways, graduates, bachelor, PhD	Connects AI to educational pipelines and workforce preparation.
Application expansion	Skills/jobs and economy/industry	77 (2.6%)/48 (1.6%)	skills, jobs, recruitment, occupation, economy, investment	Connects AI to employment and industry; not an emotion-support topic.
Application expansion	IT/assistive design	73 (2.5%)	information technology, assistive, informatics, design	Potentially relevant for support tools, but only indirectly linked to emotion regulation.
Ethics and governance	Ethics; research/government; policy	53 (1.8%)	ethics, morality, government, funded, policy, security	Relevant to privacy, bias, accountability, and human oversight.
Underdeveloped in corpus	Sport/exercise, peer interaction, campus ecology	67 (2.3%)	physical activity, sport, exercise, peer, campus appeared diffusely	A gap in the AI Index discourse; not evidence that the wider literature ignores these issues.

Note: Topic size refers to the number and percentage of non-outlier text segments assigned to each retained topic. Percentages were calculated using 2961 topic-assigned segments as the denominator, after excluding the BERTopic outlier/noise topic (−1; n = 958). Percentages are descriptive and should not be interpreted inferentially. Because Table 2 reports retained topics selected for conceptual relevance, the percentages do not sum to 100%.

**Table 3:** Practical implementation of the human–technology–environment framework.

Stakeholder	Possible Role	Safeguard	Example of Cautious Implementation
Universities	Coordinate PE, counseling, student affairs, and data governance.	Clear policy, opt-out routes, data minimization.	Use aggregated wellbeing dashboards for service planning, not individual surveillance.
PE teachers/coaches	Adapt tasks and climates in response to observed and reported needs.	Human judgment and student choice remain primary.	Offer alternative activity formats when anxiety or fatigue is detected.
Sport psychologists/counselors	Interpret emotional patterns and support referral decisions.	No automated diagnosis; professional review required.	Use AI summaries as prompts for consultation rather than as clinical conclusions.
AI designers	Build transparent, auditable, context-aware support tools.	Bias testing, explainability, limited retention, consent.	Design feedback that explains uncertainty and encourages reflection.
Students/peers	Participate in co-design and peer-support activities.	No forced disclosure of emotional data.	Create voluntary group challenges focused on enjoyment and belonging.

## Data Availability

The core corpus was derived from publicly available Stanford AI Index Reports (2021–2025). Processed materials and analytic details can be made available by the corresponding author upon reasonable request.

## Supplementary Materials

The Stanford AI Index Reports (2021–2025) analyzed in this study are publicly available from the Stanford Institute for Human-Centered Artificial Intelligence at https://hai.stanford.edu/ai-index/2026-ai-index-report (accessed on 5 July 2026) (D. Zhang et al., 2021, 2022; Maslej et al., 2023, 2024, 2025).

## Abbreviations

The following abbreviations are used in this manuscript:

Abbreviation	Meaning
AI	Artificial Intelligence
BERTopic	Bidirectional Encoder Representations from Transformers Topic Modeling
c-TF-IDF	Class-Based Term Frequency-Inverse Document Frequency
HDBSCAN	Hierarchical Density-Based Spatial Clustering of Applications with Noise
UMAP	Uniform Manifold Approximation and Projection
